# Energy-efficiency of Cardiomyocyte Stimulation with Rectangular Pulses

**DOI:** 10.1038/s41598-019-49791-w

**Published:** 2019-09-16

**Authors:** Martin Laasmaa, Pengfei Lu, Mladen Veletić, William E. Louch, Jacob Bergsland, Ilangko Balasingham, Marko Vendelin

**Affiliations:** 1 Laboratory of Systems Biology, Department of Cybernetics, School of Science, Tallinn University of Technology, 12618 Tallinn, Estonia; 2 Intervention Centre, Oslo University Hospital (OUS), 0372 Oslo, Norway; 3 Institute of Clinical Medicine, University of Oslo (UiO), 0372 Oslo, Norway; 4 Faculty of Electrical Engineering, University of Banja Luka (UNIBL), 78000 Banja Luka, Bosnia and Herzegovina; 5 Institute for Experimental Medical Research, University of Oslo, Oslo, Norway; 6 Department of Electronics and Telecommunications, Norwegian University of Science and Technology (NTNU), 7491 Trondheim, Norway

**Keywords:** Interventional cardiology, Cardiac device therapy, Cardiovascular biology, Membrane biophysics

## Abstract

In cardiac pacemaker design, energy expenditure is an important issue. This work aims to explore whether varying stimulation pulse configuration is a viable optimization strategy for reducing energy consumption by the pacemaker. A single cardiomyocyte was used as an experimental model. Each cardiomyocyte was stimulated with different stimulation protocols using rectangular waveforms applied in varying number, in short succession. The amplitude, the width of each pulse, and the interval between consecutive pulses were modified. The application of multiple pulses in a short sequence led to a reduction of the threshold voltage required for stimulation when compared to a single pulse. However, none of the employed multi-pulse sequences reduced the overall energy expenditure of cell stimulation when compared to a single pulse stimulation. Among multiple pulse protocols, a combination of two short pulses (1 ms) separated with a short interval (0.5 ms) had the same energy requirements as a single short pulse (1 ms), but required the application of significantly less voltage. While increasing the number of consecutive pulses does not reduce the energy requirements of the pacemaker, the reduction in threshold voltage can be considered in practice if lower stimulation voltages are desired.

## Introduction

The life expectancy of an implanted cardiac pacemaker is critically determined by its battery capacity. New technologies have improved battery longevity and decreased the size of the devices, which has enabled simple single chamber leadless pacemakers to function for up to 10 years. However, decreasing current drain is essential if multi-chamber leadless pacemakers with sophisticated control and communication abilities are going to be realized for long-term use^1,2^.

State-of-the-art pacemakers, including presently available leadless pacemakers, use electrodes with relatively large surface areas (diameter 6 mm). This has a direct impact on the voltage threshold and pulse duration required for reliable pacing, and thus the energy consumption of the device^3,4^. One approach to reducing energy consumption is to decrease the surface area of the stimulating electrode^5–7^. Indeed, smaller areas of the myocardium are expected to be targeted by future pacemakers, as emerging technologies enable the design and fabrication of nano-scale electrodes^8^. While such modifications may increase the efficiency of pacing, there are other trends in the industry that may limit battery capacity. For example, reduction in pacemaker size, as a means to reduce tissue damage, would imply reduction of the battery size as well. For better control of cardiac contractile synchrony, there is a desire to have multiple pacemakers in the heart which increases energy expenditure imposed by the communication protocols. Thus, alternative strategies for reducing pacemaker energy consumption per single stimulation are sought.

Pacemakers that are presently in clinical use utilize rectangular waves, sine waves, or half-sine waves to actuate the heart^9,10^. Traditionally, the stimulation consists of a single pulse, for which the duration is set as an important optimization parameter^11^. However, multi-pulse stimuli have been applied as a strategy to decrease the stimulation threshold in axons of neural cells^12^. It is possible, therefore, that applying multiple stimulus pulses in a short sequence could also reduce the required threshold stimulation voltage in the heart, and lead to a reduction of energy expenditure.

The aim of this study is to evaluate energy efficiency and threshold voltages of single- and multiple-pulse stimulation sequences for actuation of the heart. We used a single isolated cardiomyocyte as an experimental model. In the experiment, the isolated cardiomyocyte was stimulated through microelectrodes in its vicinity mimicking local pacing of the myocardium by a pacemaker. By following intracellular calcium concentration changes we were able to determine the success of each stimulation protocol and relate it to the energy employed for stimulation.

## Methods

### Cardiomyocyte isolation

Ventricular cardiomyocytes were isolated from 12 C57BL/6 mice hearts (5 male and 7 female, 135–188 days old with body weight of 28 ± 5 g) using a method based on^13^ and fully described in^14^. Briefly, the heart was excised and cannulated by the aorta on a Langendorff perfusion system after the mouse was anesthetized with a mixture of ketamine and dexmedetomidine (150 mg/kg and 0.5 mg/kg, respectively). The heart was then perfused with a wash solution until it was rinsed free of blood, followed by an enzyme-containing digestion solution. Digestion was continued until the perfusion pressure decreased to 30–40% of the initial value and the heart was soft. The ventricles were then cut into small pieces, and further incubated at 37 °C in digestion solution. During this post-digestion, cells were harvested with a Pasteur pipette, filtered through a 100 *μ*m mesh, and cells were collected in a sedimentation solution. Extracellular Ca^2+^ was then gradually increased to 2 mM to ensure Ca^2+^ tolerance of the cells. After this, extracellular Ca^2+^ was again washed out by rinsing the cells three times with sedimentation solution. The isolated cells were stored in this solution at room temperature until use.

All animal procedures were approved by the Estonian National Committee for Ethics in Animal Experimentation (Estonian Ministry of Agriculture). All experiments were performed in accordance with relevant guidelines and regulations.

### Solutions

The wash solution consisted of (in mM) 117 NaCl, 5.7 KCl, 1.5 KH_2_PO_4_, 4.4 NaHCO_3_, 1.7 MgCl_2_, 21 HEPES, 20 taurine, 11.7 glucose and 10 2, 3-butanedione monoxime. pH was adjusted to 7.4 with NaOH.

For the digestion solution, 0.25 mg/ml Liberase DL (Roche) and 1.36 mg/ml Dispase II (Roche) were added to 20 ml of the wash solution.

For the sedimentation solution, 2 mM pyruvate, 10 *μ*M leupeptin (Roche), 2 *μ*M soybean trypsin inhibitor, and 3 mg/ml BSA (Roche) were added to 40 ml of the wash solution.

For experiments, the following bath solution was used (in mM): 150 NaCl, 5.4 KCl, 0.33 NaH_2_PO_4_, 1 MgCl_2_, 1.13 CaCl_2_, 10 glucose and 10 HEPES. pH was adjusted to 7.4 with NaOH. The same solution was also used to fill the glass pipettes.

All chemicals were obtained from Sigma-Aldrich if not otherwise mentioned.

### Experimental setup

#### Electrical measurements

**Figure 1:**
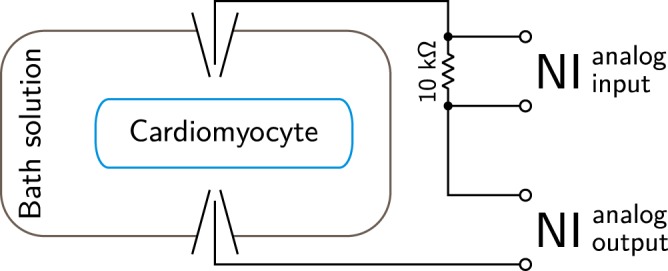
The overall scheme of the experimental setup. The cardiomyocyte was placed in the bath solution and stimulated through pipettes. The voltage between the pipettes was driven by a National Instruments data acquisition card (NI) analog output with the current estimated from a voltage drop induced by a 10 kΩ resistor. The voltage drop was measured by the NI analog input.

We used a NI PCIe-6353 data acquisition board (National Instruments, USA) for cell stimulation and applied current recordings. The principal electrical scheme is shown in Fig. 1. Two glass pipettes were filled with the bath solution and placed on opposite sides of a cardiomyocyte using micromanipulators (Scientifica, UK). The chlorinated silver wires in the glass pipettes were connected to the NI analog output port to enable cell stimulation in the range of ±10V. A 10 kΩ resistor was connected in series to allow current estimation based on the corresponding voltage drop. Data were recorded at 10 kHz using the NI-DAQmx C++ library and custom-made software.

Glass pipettes were pulled from borosilicate glass capillaries with length = 76 mm, OD = 1.5 mm, ID = 1.12 mm (TW150F-3, WPI, Sarasota, FL, USA) using a PC-10 pipette puller (Narishige, Japan). Average pipette resistance was calculated by dividing the applied voltage by the corresponding current passed during the voltage step assuming that the resistance is mainly due to the pipette tips while other components of the system (Fig. 1) are negligible. The average total resistance was 500 ± 30 kΩ (250 kΩ per pipette).

#### Fluorescence measurements

Prior to experimental recordings, each batch of cells was loaded with 1 *μ*M of the Ca^2+^ -sensitive dye Fluo-4 AM (Invitrogen), for 15–20 min at room temperature. After loading, cells where washed twice with the bath solution.

The optical scheme of the microscope is the same as described in^15^, with the optical filters and mirrors for excitation and emission described in^16^. In short, a Nikon Eclipse Ti-U microscope (Nikon, Japan) was equipped with an EMCCD camera (Andor Ixon, Andor Technologies, Belfast, UK) for rapid fluorescence measurements. The EMCCD camera image was binned 16×, gain was set to 200, and images were acquired at 170 frames per second (6 ms between frames). A CCD camera (IPX-VGA210-LMCN, Imperx Inc., FL, USA) was used to capture transmission images. Imaging was conducted via a 40× objective (CFI Super Plan Fluor ELWD 40×C NA 0.60, Nikon, Japan). Fluo-4 was excited at 482 ± 18 nm and emission was collected at 536 ± 20 nm.

### Experiments

**Figure 2:**
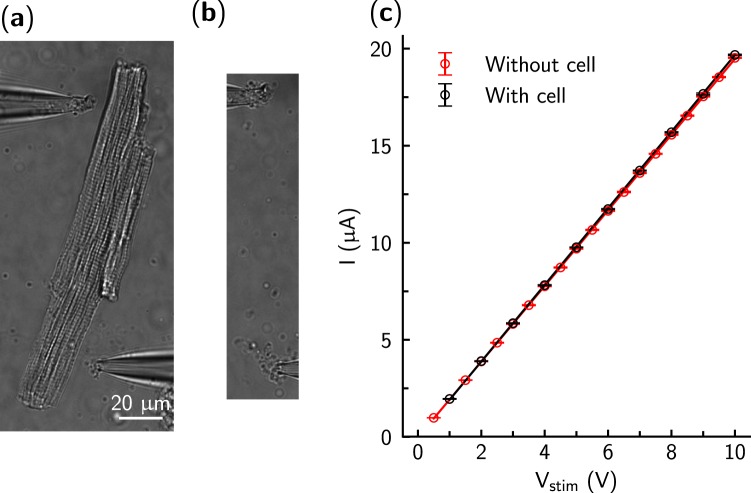
The experimental setup with (**a**) and without (**b**) a cardiomyocyte between the two pipette tips in the bath solution. The current between the pipette tips was linearly dependent on the applied voltage (**c**).

**Figure 3:**
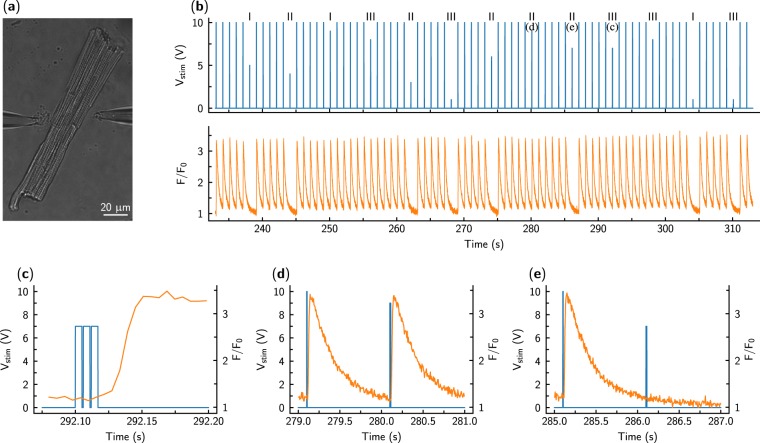
Response of a cardiomyocyte (**a**) to the stimulation by a sequence of pulses. The cardiomyocyte was excited by changes in voltage *V*_*stim*_ between the pipette tips applied as short rectangular pulses ((**b**), top). The pulse sequence consisted of 5 larger single pulses (pre-pulses, used to precondition the cell) followed by a studied stimulation sequence with varying pulse parameters. In (**b**) (top), changes in applied voltage are visible with the number of pulses per stimulation sequence marked as Roman numerals on the top of the trace. In response to the pulse, the cardiomyocyte was either stimulated or not. When stimulated, the fluorescence increase was somewhat delayed after the applied stimulation sequence (**c**), in agreement with the mechanism of Ca^2+^ -induced Ca^2+^ -release. Stimulation was assessed by recording fluorescence *F* of the Ca^2+^ sensitive dye and is shown normalized to the resting cellular fluorescence *F*_0_. Note that while the preconditioning pulses always elicited a Ca^2+^ transient, the success of cellular activation was variable for the studied pulses. As examples, stimulated (**d**) and non-stimulated (**e**) cases are shown with the preceding preconditioning pulse (see subplot markings at the top of the trace in (**b**) next to the corresponding pulse). In the figure, voltage and fluorescence traces are shown in blue and orange, respectively.

Each batch of cells was placed under the microscope in the RC-49FS chamber (Warner), containing the bath solution. Cells were allowed to attach to the coverslip for ~5 min. A single cell was then carefully approached with two glass pipettes, which were positioned on opposite sides of the cell as shown in Fig. 2(a), and as close as possible to the coverslip. Typically, the pipette tips were placed roughly 5–10 *μ*m from the cell membrane and the distance between the pipette tips was in the range of 30–130 *μ*m (80 ± 25 *μ*m, mean ± SD). Here, the actuation of the cell depends on the strength of the electrical field as well on cell’s position in the field. As the strength of the electrical field between two pipette tips is inversely proportional to the distance between them, maximal field strength can be achieved when the tips are closest together at opposite sides of a cell (similar to the situation show in Fig. 3(a)). However, with this orientation the portion of the cell’s membrane that is effectively affected by the field is minimal. In practice, moving the pipettes apart from each other along the cell’s length (Fig. 2(a)) reduced the voltage needed for cell actuation.

Next, a minimal voltage of a single rectangular pulse was found that stimulated the cell. This voltage was scaled up by 10–20% and used as the preconditioning pulse amplitude (*V*_*p*_). A stimulation sequence was then applied consisting of 100 preconditioning rectangular pulses with amplitude = *V*_*p*_, pulse width = *w*, and time between pulses = 1 s. Note that the current between pipette tips was linearly dependent on applied voltages, irrespective of the presence of a cardiomyocyte between the pipette tips, as shown in Fig. 2(c).

After the initial stimulation with 100 pulses, the main experiment was performed. Before each test pulse sequence, five pre-pulses were applied with the same amplitude *V*_*p*_ as the preconditioning pulses described above. Each stimulus combination was repeated at least three times during a single experiment. The order of stimulation pulse sequences was randomized to avoid bias.

The stimulation pulse test sequences consisted of either 1, 2 or 3 sequential rectangular pulses with amplitude in the range from 1 to 10V (1V step) and pulse width *w*. These pulses were separated by a resting interval Δ. Within each experiment, pulse width *w* and the interval between sequential stimulus pulses Δ were kept constant. Duration of a single experiment was 600–900 s.

All recordings were conducted at room temperature (23 °C, temperature controlled by the air conditioner).

### Data analysis

Data from the background area was subtracted to find cellular fluorescence (*F*). In the following analysis, *F* was always normalized by the resting cellular fluorescence (*F*_0_).

For determination of the voltage corresponding to the stimulation threshold, we fitted the normalized fluorescence amplitude *f* with the Hill equation:

1 $$f(V)=A\cdot \frac{{V}^{h}}{({V}_{50}^{h}+{V}^{h})}+B,$$

where *A* is fluorescence amplitude (in *F*/*F*_0_), *V* is the voltage applied, *h* is the Hill coefficient, *B* is offset, and *V*_50_ is the half-saturation constant that was considered to be the threshold voltage. Here, the equation was used to fit the data phenomenologically to reproduce the sharp transition from the non-actuated to actuated state. Only *V*_50_ values were used in the analysis. The energy expenditure at *V*_50_ was calculated as:

2 $${E}_{50}={V}_{50}^{2}\cdot w\cdot n,$$

where *w* is the pulse width, and *n* is the number of sequential rectangular pulses in the stimulus.

To compare data recorded from different cells, we normalized *V*_50_ and *E*_50_ obtained from the stimulation sequences with multiple pulses (*n* = 2 or 3) to the respective values (*V*_50*n*1_ and *E*_50*n*1_) obtained from single pulse stimulation (*n* = 1). This was done to avoid cell-to-cell variability in the positioning of the pipettes.

### Statistics

If not stated otherwise, statistics is shown as mean ± standard deviation. An exception is the case, where Tukey box plots are presented; the horizontal line in each box shows the mean, box ends show the quartiles *Q*_1_ and *Q*_3_, and whiskers extend the box ends by 1.5 times of interquartile range (*Q*_3_ − *Q*_1_). Statistical tests were performed with JASP^17^, using pairwise t-tests, one sample t-tests, and repeated measures ANOVA tests.

## Results

A typical experimental recording from an isolated cardiomyocyte stimulated by the pair of electrodes is shown in Fig. 3. As demonstrated in Fig. 3(b), preconditioning pulses always resulted in action potentials, as indicated by the triggering of Ca^2+^ -transients. A zoom-in of one particular excitation sequence in Fig. 3(c) shows the rise in Ca^2+^ fluorescence following the end of the stimulus train. Depending on the stimulation sequence configuration, some stimuli resulted in action potentials Fig. 3(d) and some did not Fig. 3(e). Thus, there is a certain threshold voltage which must be achieved to actuate a cell.

**Figure 4:**
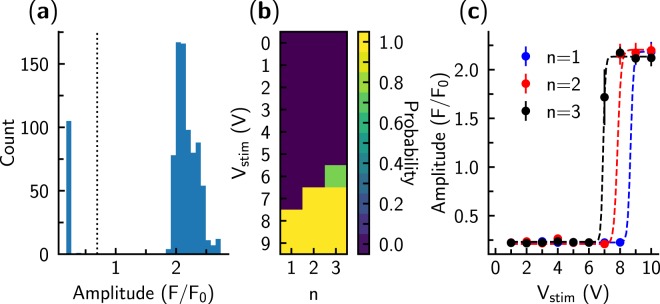
Analysis of the recordings performed on a single cardiomyocyte. (**a**) Fluorescence changes during a single period were analyzed by following the amplitude of the changes. Note that the distribution of the amplitudes was bimodal: one population corresponded to cases with successful stimulation of the cell (larger *F*/*F*_0_) and one to unsuccessful stimulation (lower *F*/*F*_0_). The threshold value used for detection of cell stimulation by the given pulse is shown as a dashed line. (**b**) Average stimulation success rate for the pulses with different voltages *V*_*stim*_ and number of pulses *n* shown by color. Here, each pulse sequence was applied 5 times during the experiment. Note that for most of the pulse sequences the stimulation was either always or never successful (probabilities 0 and 1, respectively). However, for a triple pulse with *V*_*stim*_ = 6V, one of the 5 tests did not induce stimulation. (**c**) Threshold voltage for each of the pulse sequences was found by fitting the Hill equation to the average fluorescence amplitude when the fluorescence was studied as a function of the pulse voltage. Note that lower voltages were required to stimulate the cell if there were more pulses per each stimulation sequence (*n*). As an example, for this particular case, fitted values for *n* = 1 were *A* = 1.96, *B* = 0.22, *V*_50_ = 8.67, and *h* = 91.84. Here, the Hill equation is used only as a formal equation for description of transition from one state to another.

To find this threshold voltage, we determined whether the cardiomyocyte was stimulated at each applied voltage step. For this, we observed that the distribution of fluorescence amplitudes had two distinct peaks: one corresponding to the periods where cardiomyocytes were stimulated, and one corresponding to the periods without successful stimulation where the Ca^2+^ responses were smaller (Fig. 4(a)). This clear separation allowed us to determine the success of each stimulation using a simple threshold between the two peaks. Since each stimulation sequence was repeated several times, we were able to determine the probability of actuation of the cell (Fig. 4(b)).

For determination of the voltage corresponding to the stimulation threshold, we fitted the fluorescence amplitude with Eq. (1) and used the half-saturation constant as a threshold (Fig. 4(c)). As shown in Fig. 4(c), the threshold voltage was lower when the number of pulses *n* was increased compared to a single pulse stimulation.

**Figure 5:**
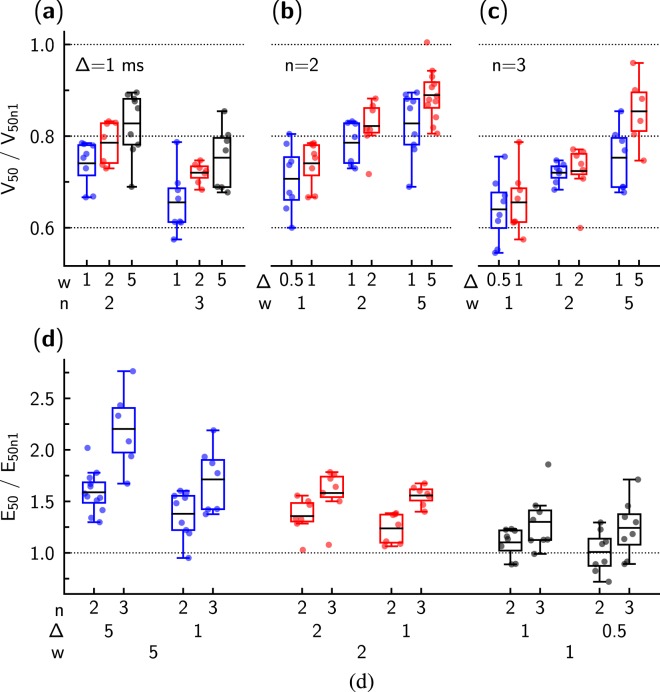
Statistical analysis of the recorded data. Threshold voltage *V*_50_ and energy expenditure E_50_ obtained for the stimulation sequences with the multiple pulses (*n* = 2 or 3) were normalized to the respective values obtained for the same pulse width *w* (shown in ms) and inter-pulse interval Δ (shown in ms) for a single pulse stimulation (*n* = 1). (**a**–**c**) show threshold voltage *V*_50_ dependency on stimulation pulse width *w*, the number of pulses used in the sequence *n*, and the inter-pulse interval Δ. In (**a**), at the same interval Δ, the influence of pulse width *w* and the number of pulses *n* is demonstrated. In (**b**,**c**), note that multiple pulses with smaller *w* could lead to a significant reduction in the threshold voltage (*V*_50_/*V*_50*n*1_ < 1). In (**d**), energy expenditure E_50_ required to activate cardiomyocyte is shown for studied pulse sequences. For most of the sequences examined, E_50_ was larger when the cardiomyocyte was stimulated using multiple pulses (*n* > 1). An exception to this rule was the double pulse with *w* = 1 ms and Δ = 0.5 or 1 ms.

To compare the performance of different stimulation sequences, threshold voltages were normalized to the threshold voltage obtained from the single pulse protocol. As shown in Fig. 5, threshold voltages were reduced with an increased number of pulses. For statistical analysis, we first compared threshold voltages for two- or three-pulse stimuli with the threshold voltage for single pulse stimulation in the same cell (paired samples t-test). Pooled data showed a statistically significant reduction in threshold voltage (*p* < 0.001) for both comparisons (*V*_50*n*1_ − *V*_50*n*2_ and *V*_50*n*1_ − *V*_50*n*3_). Normalized threshold voltages *V*_50*n*2_/*V*_50*n*1_ and *V*_50*n*3_/*V*_50*n*1_ were also significantly lower than one (*p* < 0.001, one sample t-test, all data pooled together).

The reduction of the threshold voltage depended on the duration of a single pulse in the sequence (Fig. 5(a)). By comparing the different sequences, we observed that the voltage reduction obtained with multiple pulses tended to be more pronounced when the pulses were shorter. For example, when comparing the voltage changes observed for the sequences with the interval between pulses Δ = 1 ms (Fig. 5(a)), a significant effect of pulse width *w* was detectable (*p* = 0.01, repeated measures ANOVA). In this test, *V*_50*n*2_/*V*_50*n*1_ and *V*_50*n*3_/*V*_50*n*1_ were within-subject measurements (effect with *p* < 0.001), while pulse width *w* was varied between cells.

The effect of the interval between pulses on the threshold voltages was more pronounced for larger Δ (Fig. 5(b,c)). The reduction in the threshold voltage did not change significantly by increasing the interval Δ from 0.5 to 1 ms (1 ms wide pulses, *p* = 0.5, repeated measures ANOVA), but the difference approached significance for comparison between Δ = 1 ms and Δ = 5 ms intervals (*w* = 5 ms pulses, *p* = 0.07, repeated measures ANOVA). In these tests, *V*_50*n*2_/*V*_50*n*1_ and *V*_50*n*3_/*V*_50*n*1_ were within-subject measurements (effect with *p* < 0.001), while Δ was varied between cells.

After establishing that multiple pulse stimulation allowed reduction of threshold voltage, energy expenditure E_50_ was calculated (Eq. (1)) for the studied pulse sequences (Fig. 5(d)). As with threshold voltage, we normalized E_50_ to the single pulse E_50_. As shown in Fig. 5(d), in general, the use of multiple pulse stimuli required more energy to excite a cardiomyocyte than the use of single pulse stimulus. The only exceptions from this general behavior were sequences employing short pulses (*w* = 1 ms) that were separated by a short interval (Δ = 0.5 ms or 1 ms). Specifically, when this pulse was applied twice (*n* = 2), E_50_ was the same for single and double-pulse sequences (*p* = 0.95, paired t-test, testing E_50*n*2_ − E_50*n*1_; *p* = 0.86, testing E_50*n*2_/E_50*n*1_ against one). When three pulses were used in the sequence, E_50_ was already significantly larger than for single pulse stimulation (*p* < 0.05, paired t-test, testing E_50*n*2_ − E_50*n*1_). However, none of the studied multipulse sequences were able to excite a cardiomyocyte with lower E_50_ than a single pulse.

A summary of recorded data changes in threshold voltage and energy expenditure E_50_ is shown in Table 1.

**Table 1:** Summary of the recorded data.

*w* (ms)	Δ (ms)	No. of cells	*V*_50_/*V*_50*n*1_		*E*_50_/*E*_50*n*1_	
			*n* = 2	*n* = 3	*n* = 2	*n* = 3
			mean ± SD	mean ± SD	mean ± SD	mean ± SD
1	0.5	8	0.71 ± 0.07^#^	0.64 ± 0.07 #	1.01 ± 0.20 ND	1.24 ± 0.27^*^
	1	8	0.74 ± 0.05^#^	0.66 ± 0.07 #	1.10 ± 0.14 ND	1.30 ± 0.28^*^
2	1	7	0.79 ± 0.05^#^	0.72 ± 0.02 #	1.24 ± 0.15**	1.56 ± 0.10^#^
	2	7	0.82 ± 0.06^#^	0.72 ± 0.06 #	1.36 ± 0.18**	1.58 ± 0.24^#^
5	1	9	0.83 ± 0.07^#^	0.75 ± 0.07 #	1.38 ± 0.23^#^	1.71 ± 0.31^#^
	5	12/6	0.89 ± 0.06^#^	0.85 ± 0.08 **	1.59 ± 0.20^#^	2.20 ± 0.39^#^

Mean values ± standard deviation are presented for normalized threshold voltage (*V*_50_/*V*_50*n*1_) and energy expenditure (*E*_50_/*E*_50*n*1_) from the stimulation sequences with multiple pulses (*n* = 2 or 3), relative to single pulse (*n* = 1). Measurements were made at fixed pulse width *w* and interval Δ between the pulses. Comparison with *n* = 1 pulses was done with one-sample t-test against value 1. Effect size shown as follows: ND - no difference; *p < 0.05; **p < 0.01; ^#^*p* < 0.001. Note that in the case *w* = 5 and Δ = 5 the number of cells used in statistics was 12 and 6 for *n* = 2 and 3, respectively.

## Discussion

The main result of our study is that electrical stimulation with multiple rectangular pulses does not lead to energy savings in cardiomyocyte stimulation. However, the use of multiple short pulses enables reduction in the stimulation threshold voltage. Indeed, when the pulses are sufficiently short and the inter-pulse interval is brief, energy expenditure is equivalent to that produced during a single pulse stimulation.

When analyzing the obtained threshold voltages and energy expenditure, it is critical to consider that the experiments were performed on single cardiomyocytes. *In vivo*, the employed stimulation pattern would be applied to the region of the myocardium that is in the vicinity of the pacemaker leads, and all nearby cardiomyocytes would be exposed to the stimulus at the same time. By comparison, our present experiments only examine the stimulation property of a single cardiomyocyte without the effect of the load imposed by the neighboring tissue. However, we expect that the general properties would remain the same for the tissue stimulation as for the single cardiomyocyte when the pulse sequences and their properties are analyzed. Nevertheless, it is clear that the load on cells stimulated *in vivo* could change the absolute values of the optimal pulse width, amplitude, and the duration between the pulses in the stimulation sequence. To obtain these optimal values, a study taking into account the geometry of pacemaker leads and interaction between leads and the tissue would have to be undertaken.

We have detected the activation of the cardiomyocyte using calcium-dependent fluorescence. An alteranative approach would be to employ a voltage-sensitive dye such as Di-8-ANEPPS. However, a key advantage of monitoring calcium-dependent fluorescence is the high sensitivity of dye employed. Indeed, larger changes in Ca^2+^ signals can be recorded from the whole cell, rather than voltage recordings which are only made at the cell membrane. Moreover, by following calcium-induced fluorescence changes, we ensured that we registered only those stimuli that resulted in full activation as being successful.

Our study employed mouse cardiomyocytes at room temperature. Mice are a routinely used animal model, and ventricular cardiomyocytes from murine hearts have similar action potential upstroke characteristics as in human cardiomyocytes^18^. The temperature was selected due to the technical limitations of the setup, and should be taken into account when interpreting the experimental data through mathematical modeling. While cardiomyocytes were stable during recordings, we did not expose each cardiomyocyte to all considered pulse forms to keep the experimental time relatively short. In particular, we limited the sequence of the studied pulses to maintain a fixed pulse width *w* and inter-pulse interval Δ for each of cell. This protocol was tailored toward a comparison of single-pulse and multiple-pulse stimuli, in accordance with our aim. However, as a result, we cannot analyze how single-pulse activation depends on pulse width, for example, since this would require measurements from different cells. As the cell positioning and applied electrical field differed between experiments, such comparisons cannot be made on the basis of our data.

In this work, we used only rectangular pulses for stimulation and have not examined possibilities for other, energetically favorable pulse shapes^19,20^. Since all experiments must be performed while the cells were viable, the imposed time constraints made it impossible to test a variety of waveforms. By limiting our study to a single waveform, we were able to analyze the properties of multiple pulse stimulation within the same cell and with the same positioning of the electrodes. As a result, we were able to minimize cell-to-cell variability and variability induced by the electrodes which could have reduced the resolving power of the analysis. We were able to demonstrate that while the threshold voltage is reduced when multiple pulses are used, the energy expenditure per successful activation of the cell is optimal when using a single pulse stimulation.

The threshold voltage applied to the cardiomyocyte in our experiments is dependent on the electrical field surrounding the cell and induced by the voltage applied through the pipettes. To reduce effects due to variability in the placement of the pipettes and cell shape, we determined the threshold voltage, and normalized recordings to this value. Due to the linear relationship between the current and voltage (Fig. 2) and applied normalization, we could statistically analyze the recordings obtained from different cells.

For each of the cells, we repeated the same waveform multiple times. As demonstrated in Fig. 4(c), the results were very reproducible throughout the whole experiment. In agreement with such stability, the resistance between pipettes remained consistent throughout the experiment (changes were 0.9 ± 0.5% when the beginning and the end resistance were compared). Thus, there was no significant debris buildup that might influence our results.

While rectangular pulses were used, we would expect that the outcome using optimal pulse configurations^19,20^ would be similar if single and multiple pulses were compared as done here. There is a significant difference between the analysis of optimal pulse form and application of multiple pulses to the cell, as done here. In particular, in contrast to^19^, we did not assume that there is a threshold potential that must be attained for activation. In our work, we looked into whether application of multiple pulses would precondition the cardiomyocyte into a state where it is possible to activate it with a smaller energy expenditure. As we did not, in fact, observe such a reduction in energy expenditure, our results are consistent with the membrane acting as a capacitor. As such, a threshold must be reached to initiate a cascade of events leading to stimulation of a cardiomyocyte. Due to the leaky nature of the membrane, induced by currents that maintain membrane potential at the resting state, the energy expenditure increases if there is a significant delay between stimuli.

While we did not observe a reduction of stimulation energy expenditure, the use of multiple pulses, such as doublets, could allow reduction of stimulation voltage. As shown in our results, application of pulses separated with a short interval, would reduce the voltage that must be applied for stimulation of a cardiomyocyte. Such a reduction may be beneficial, by minimizing the effects of cardiac pacing on the pectoral muscles^21^ and diaphragm^22^. Thus, in addition to pulse voltage and pulse duration optimization^11^, fine tuning of the number of stimulus pulses could provide a means for optimizing cardiac pacemaking when side effects are taken into account.

## Data Availability

The datasets generated during the current study are available from the corresponding author on reasonable request.
